# Placental Neutrophil Infiltration Associated with Tobacco Exposure but Not Development of Bronchopulmonary Dysplasia

**DOI:** 10.3390/children9030381

**Published:** 2022-03-09

**Authors:** David M. Box, Abhishek Makkar, Zhongxin Yu, Hala Chaaban, Henry H. Tran, Kathryn Y. Burge, Jeffrey V. Eckert

**Affiliations:** 1 Neonatal-Perinatal Medicine, Department of Pediatrics, University of Oklahoma Health Sciences Center, Oklahoma City, OK 73104, USA; davidbox22@gmail.com (D.M.B.); abhishek-makkar@ouhsc.edu (A.M.); hala-chaaban@ouhsc.edu (H.C.); kathryn-burge@ouhsc.edu (K.Y.B.); 2 Pathology Department, University of Oklahoma Health Sciences Center, Oklahoma City, OK 73104, USA; zhongxin-yu@ouhsc.edu (Z.Y.); hanh-tran@ouhsc.edu (H.H.T.)

**Keywords:** bronchopulmonary dysplasia, preterm, maternal tobacco exposure, maternal smoking neutrophil, neutrophil gelatinase-associated lipocalin (NGAL)

## Abstract

Objective: In utero inflammation is associated with bronchopulmonary dysplasia (BPD) in preterm infants. We hypothesized that maternal tobacco exposure (TE) might induce placental neutrophil infiltration, increasing the risk for BPD. Study design: We compared the composite outcome of BPD and death in a prospective pilot study of TE and no-TE mothers and their infants born <32 weeks. Placental neutrophil infiltration was approximated by neutrophil gelatinase-associated lipocalin (NGAL) ELISA, and total RNA expression was analyzed via NanoString© (Seattle, WA, USA). Result: Of 39 enrolled patients, 44% were classified as tobacco exposure. No significant difference was noted in the infant’s composite outcome of BPD or death based on maternal tobacco exposure. NGAL was higher in placentas of TE vs. non-TE mothers (*p* < 0.05). Placental RNA analysis identified the upregulation of key inflammatory genes associated with maternal tobacco exposure. Conclusion: Tobacco exposure during pregnancy was associated with increased placental neutrophil markers and upregulated inflammatory gene expression. These findings were not associated with BPD.

## 1. Introduction

Tobacco exposure (TE) during pregnancy is highly prevalent in the United States. As reported by the Center for Disease Control and Prevention (CDC) in 2016, 7.2% of mothers smoked cigarettes during pregnancy [1]. It is well recognized that maternal tobacco use during pregnancy is linked to many negative outcomes for infants, including low birthweights, preterm birth, preterm prolonged rupture of membrane (PPROM), and other birth defects [2,3,4,5].

Recently, Antonucci et al. indicated that in utero exposure to smoking is an independent risk factor for the development of bronchopulmonary dysplasia (BPD) in premature infants born weighing less than 1500 g [6]. BPD is the most prevalent sequela of preterm birth, affecting 10,000–15,000 infants annually in the United States [7]. Known postnatal risk factors for the disease include hyperoxia, mechanical ventilation, patent ductus arteriosus (PDA), and sepsis; antenatal risk factors include chorioamnionitis, preeclampsia, and hypertension [8,9,10,11,12].

Neutrophil gelatinase-associated lipocalin (NGAL) is a glycoprotein found predominantly in neutrophil granules. NGAL is normally expressed at low levels but is often elevated in the blood, bronchoalveolar lavage (BAL) fluid, and sputum in adults with lung diseases, such as asthma and chronic obstructive pulmonary disease (COPD) [13]. Notably, serum levels of NGAL at birth are significantly higher in preterm infants who develop BPD than in those who do not [14], suggesting a potential role for NGAL as a biomarker for BPD.

Little is known about the mechanism by which maternal tobacco exposure is associated with the development of BPD. A previous study demonstrated a higher number of neutrophils within the placentas of mothers who smoked during pregnancy; however, the incidence of bacterial infection in that group was higher, confounding the results [15]. Recent reviews have focused on injury and its contribution to fetal lung development, identifying inflammation and tobacco exposure as major contributors [16]. In addition, a meta-analysis of tobacco smoking during pregnancy showed significant association with BPD at a postmenstrual age of 36 weeks [17]. Finally, the adverse effects of maternal tobacco exposure are supported by epidemiological and animal studies, which demonstrate disrupted pulmonary development [18,19,20]. These observations taken together establish a link between maternal tobacco exposure and BPD and raise the possibility that neutrophils play a key role in the mechanism, with elevated levels in preterm infants who develop BDP.

To further understand the effect of antenatal tobacco exposure and its association with the development of BPD, we compared placental and infant characteristics of tobacco exposure and non-tobacco exposure mothers. Our hypothesis is two-fold; (1) we hypothesized that maternal tobacco exposure would result in increased inflammatory neutrophil infiltration of the placenta of preterm infants <32 weeks gestation, and (2) we hypothesize that infants <32 weeks gestation with tobacco exposure will subsequently be at increased risk for developing BPD. Therefore, we sought to achieve two aims/objectives in our study. The first was to identify increased neutrophil infiltration in the placenta of mothers with tobacco exposure (primary outcome). The second was to follow these infants for the composite outcome of BPD or death (secondary outcome).

## 2. Materials and Methods

**Figure A1:**
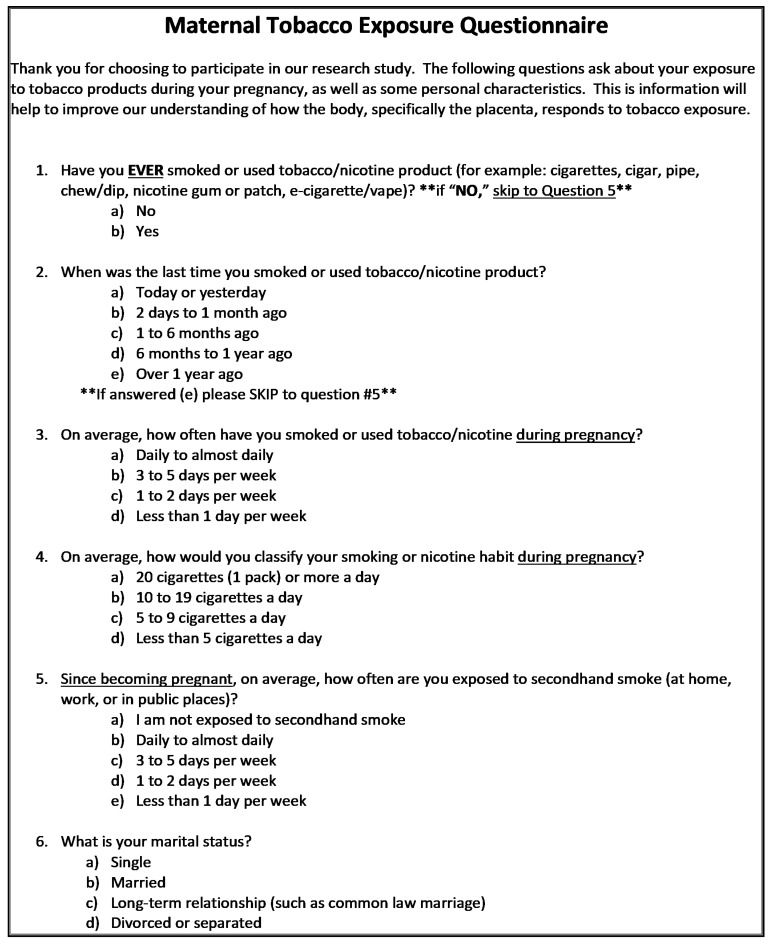

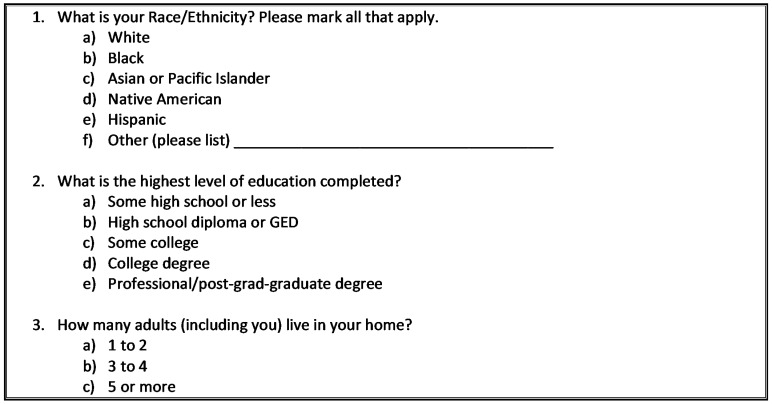
Following consent, a maternal tobacco exposure questionnaire was administered within 24 h of delivery to self-identify TE during pregnancy.

Study design: This pilot prospective, observational study was conducted between October 2018 and December 2019 and was approved by the Institutional Review Board at the University of Oklahoma Health Sciences Center (OUHSC). Written informed consent was obtained for the mother and newborn either prior to delivery or within 24 h post-delivery. Following consent, a 9-item maternal questionnaire for self-identification of tobacco exposure during pregnancy was completed (Figure A1). Our maternal questionnaire on tobacco use was internally validated in a previous study, where cotinine levels (a nicotine metabolite) were detectable only in mothers who reported tobacco exposure [21]. Patients were stratified into two groups: TE mothers and non-TE mothers.

Study population: Participants included mothers and their preterm infants born at a gestational age of <32 weeks. Infants were excluded based on known major congenital anomalies, maternal concern for infection (e.g., clinical chorioamnionitis), maternal fever >38 °C 24 h before delivery, presence of meconium-stained fluid, maternal history of impaired immunity, or a concomitant medical condition impacting inflammatory response.

Data collection: Data were de-identified and prospectively collected and managed using a data collection sheet at OUHSC. Maternal and neonatal demographic characteristics were collected via chart review. The secondary outcome was a composite of BPD or death endpoints. BPD status was assessed at 36 weeks postmenstrual age (PMA) using the National Institutes of Health (NIH) workshop definition [22]. Mild BPD is defined as breathing room air at 36 weeks corrected or time of discharge, moderate BPD is defined as needing <30% oxygen at 36 weeks corrected/discharge, whereas severe BPD is defined as needing >30% O2 at 36 weeks corrected age/discharge. For the purpose of this study, infants were defined as having the presence or absence of BPD; absence of BPD was defined as no or mild BPD, and the presence of BPD was defined as moderate to severe BPD [22]. Additional outcomes included necrotizing enterocolitis (NEC), intraventricular hemorrhage (IVH), retinopathy of prematurity (ROP), PDA, and sepsis. A mother was considered to have received antenatal corticosteroids if she received a full or partial betamethasone or dexamethasone course. Intrauterine growth restriction (IUGR) was defined as intrauterine estimated fetal weight less than the 10th percentile. PPROM was defined as having membranes ruptured for more than 18 h. Samples from the placenta from both groups were evaluated for histological chorioamnionitis by one of two pathologists blinded to maternal tobacco exposure status. Positive tobacco exposure was defined as maternal ‘daily’ to ‘almost daily’ active smoking or ‘daily’ to ‘almost daily’ secondhand smoke exposure, as reported on the maternal tobacco exposure questionnaire (Figure A1).

To determine the contribution of tobacco exposure to the development of BPD, the groups were further subdivided into (1) TE mothers with infants developing BPD (BPD TE group); (2) non-TE mothers with infants developing BPD (BPD No TE group); (3) TE mothers with infants not developing BPD (No BPD TE group); and (4) non-TE mothers with infants not developing BPD (No BPD No TE group).

Sample collection: Fresh placenta tissue samples were collected within 24 h of delivery. Three full-thickness sections of placenta parenchyma (including fetal and maternal surfaces), one section of extraplacental membrane roll, and two sections of the umbilical cord (proximal and distal) were collected and fixed in 10% formalin for routine histopathological examination and diagnosis. One full-thickness section was split and preserved for both RNA analysis (RNAlater™, Invitrogen, Carlsbad, CA, USA) and protein analysis (snap-frozen in liquid nitrogen). All samples were stored at −80 °C until further analysis.

Immunohistochemistry (IHC): IHC was performed according to the manufacturer’s protocols using a Leica Bond-IIITM Polymer Refine Detection System (DS 9800). Formalin-fixed paraffin-embedded (FFPE) tissues were sectioned at the desired thickness (4 µm) and mounted on positively charged slides. The slides were dried overnight at room temperature and incubated at 60 °C for 45 min, followed by deparaffinization and rehydration in an automated multi-stainer (Leica ST5020). Subsequently, slides were transferred to the Leica Bond-IIITM and treated for antigen retrieval at 100 °C for 20 min in a retrieval solution, at either pH 6.0 or 9.0. Endogenous peroxidase was blocked using a peroxidase-blocking reagent, followed by 60 min of incubation with NGAL antibody (Catalog #711280, ThermoFisher Scientific, Waltham, MA, USA) diluted 1:100. Post-primary IgG-linker and/or poly-HRP IgG reagents were used as the secondary antibody. Detection was accomplished via the chromogen 3,3′-diaminobenzidine tetrahydrochloride (DAB), and counterstained with hematoxylin. Completed slides were dehydrated (Leica ST5020) and mounted (Leica MM24). The antibody-specific positive control and negative control (omission of primary antibody) were parallel stained. Additionally, two pathologists blinded to smoking and BPD status semi-quantitatively scored based on anatomical location, with scores from zero to four: score ‘0′ signifying no staining; score ‘1′ for 1–10 positive cells/per high power field (HPF); score ‘2′ for 11–50 positive cells/HPF; score ‘3′ for 51–75 positive cells/HPF; and score ‘4′ for >75/HPF.

Protein analysis and enzyme-linked immunosorbent assay (ELISA): ELISA was used to quantify NGAL (Catalog #036RUO, BioPorto Diagnostics A/S, Hellerup, Denmark) following the manufacturer’s instructions. Briefly, frozen placental tissue was mechanically homogenized using a BeadBeater (Next Advance Inc., Troy, NY, USA) in a buffer containing phosphatase, protease inhibitors (Catalog #524625 and #535140, Millipore, Burlington, MA, USA) and PMSF (Sigma-Aldrich, St. Louis, MO, USA). Results were normalized to total protein concentration determined by bicinchoninic acid (BCA) assay (Catalog #23227, Pierce Biotechnology, Rockford, IL, USA).

Total RNA analysis/NanoString©: A random subset of 12 patients from the four subgroups (n = 3/group): BPD, TE group; BPD, no TE group; no BPD, TE group; and no BPD, no TE group. A BeadBeater was used to homogenize placental tissue mechanically. Total RNA was extracted per the manufacturer’s protocols using a Zymo Quick-RNA MidiPrep kit (Catalog #R1056, Zymo Research, Irvine, CA, USA). Total RNA, between 25 ng and 300 ng, was loaded onto a nCounter^®^ Human Immunology v2 Panel (Catalog #XT-CSO-HIM2-12, NanoString, Seattle, WA, USA). This panel consisted of 594 genes of interest and 15 internal reference genes. Data were analyzed using nCounter Analysis and nCounter Advanced Analysis software. RCC output files were imported into NanoString nSolver 4.0. Default quality control (QC) settings were used to verify the quality of all data (>95% of fields of view [FOV] and binding densities between 0.2 and 0.5). The background was corrected by subtracting the mean value of 8 engineered RNA negative control sequences from the raw counts of all genes. The geometric mean was calculated for the 15 housekeeping genes, and the nine genes with the lowest coefficient of variation were used to normalize the data. Genes with mean normalized counts of less than 50 were excluded from the analysis. The control group was defined as No TE or No BPD No TE for subgroup analysis. Gene expressions are estimated to have a log2-fold change, holding all other variables constant. The 95% confidence intervals (CI) for the log2-fold change and the *p* values are reported. A 1.2-fold change was selected as the differential threshold.

Given the unpredictable nature of preterm deliveries, we allowed up to 24 h for placenta collection. Once collected, the placenta was immediately placed at 4 °C. The pathologist then collected full-thickness sections and stored these at −80 °C or preserved with RNAlaterTM. Although we allowed up to 24 h for placenta collection in our protocol, the majority of samples were collected within 2–12 h. This methodology allows for collection of high-quality RNA from placentas stored at 4 °C or even room temperature for up to 48 h prior to being transferred to stabilizing solution, such as RNAlaterTM [23].

Statistical methods: Our study is a pilot/preliminary study on a topic where there is little known on the association between inflammation within the placenta and development of BPD in preterm neonates. While we have directional hypotheses, we felt it would be inappropriate to quantify an effect size given the paucity of research on the topic. Descriptive statistics were computed for demographic and clinical variables. Comparisons of categorical variables between patients developing BPD or death and those who did not were evaluated with Fisher’s exact test. Continuous variables were assessed for normality, then compared between groups using a Kruskal–Wallis test or Student’s *t*-test, as appropriate. Frequencies and percentages were reported for categorical variables across BPD status. Count means and standard deviations are reported for continuous variables. Statistical significance is defined, in all experiments, as *p* < 0.05.

## 3. Results

In total, 95 mothers were screened, and 49 mothers were approached for study enrollment based on the inclusion and exclusion criteria. Eight mothers declined and two approached mothers aged out of this study (delivered baby >32 weeks gestation). Demographic characteristics for the remaining 39 patients were stratified by the presence and absence of tobacco exposure (Table 1), as well as by the presence or absence of the composite outcome of BPD or death (Table A1). Of enrolled mothers, 43.6% reported tobacco exposure during pregnancy (Table 1 and Table A2). Of these tobacco exposure mothers, two reported the exposure was via secondhand smoke.

No differences in birth weight, birth length, head circumference, gestational age, gender, maternal ethnicity, antenatal steroid, mode of delivery, intubation in delivery room, intubated in NICU, PDA medical or surgical treatment, IVH grade 3 or 4, ROP, IUGR <10th percentile, or death or BPD were noted with maternal tobacco exposure. There was an association with maternal age (*p* = 0.048), with tobacco exposure mothers being slightly older (Table 1). When comparing tobacco exposure mothers, no differences in diabetes status, maternal hypertension, prolong rupture of membranes, chorioamnionitis, antepartum hemorrhage, marijuana, or other illicit drug use were present (Table 2). No differences in the incidence of NEC, or sepsis based on maternal tobacco exposure were noted.

As expected, infants with the composite outcome of BPD or death had significantly lower (*p* < 0.001) birth weight, length, head circumference, and gestational age compared with the No BPD group. Additionally, more infants in the composite outcome required intubation in the delivery room (*p* = 0.001) or the NICU (*p* < 0.001), required medical management of PDA (*p* = 0.01), and developed threshold ROP (*p* = 0.017) compared to the No BPD group (Table A1). The remainder of the maternal and neonatal demographic characteristics did not differ between groups. From the maternal perspective, we found no significant association between tobacco exposure status and maternal complications, with the exception of increased incidence of antepartum hemorrhage in the composite outcome group (*p* = 0.003) (Table A2).

**Figure 1:**
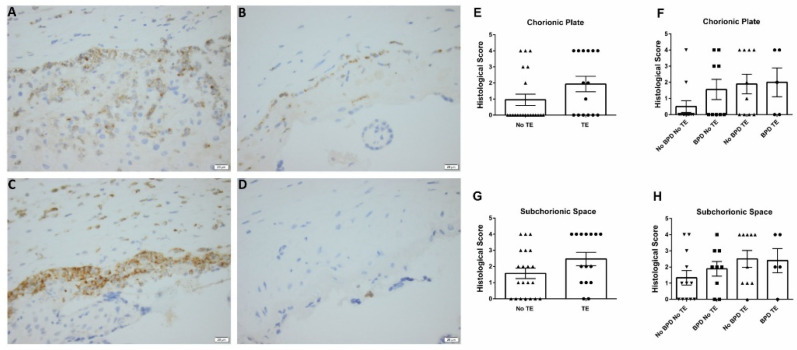
Representative diaminobenzidine (brown) and hematoxylin (blue) staining (**A**–**D**, 400X magnification), and staining quantification (**E**–**H**) for placental NGAL. (**A**) BPD, TE group (n = 5), with strong (3 to 4+) NGAL-positive staining in a chorionic plate and subchorionic space. (**B**) BPD, no TE group (n = 9), with mild (1–2+) NGAL-positive staining only in subchorionic space. (**C**) No BPD, TE group (n = 10), with strong (3–4+) NGAL-positive staining in the chorionic plate and subchorionic space. (**D**) No BPD, no TE group (n = 12), with rare (0–1+) NGAL-positive staining only in subchorionic space. (**E**,**F**) Quantification of chorionic plate staining stratified by maternal smoking status and subgroup analysis. (**G**,**H**) Quantification of subchorionic space staining stratified by maternal smoking status and subgroup analysis. NGAL—neutrophil gelatinase-associated lipocalin, BPD—bronchopulmonary dysplasia, and TE—tobacco exposure.

While there was no association between maternal tobacco exposure and an infant’s risk for developing BPD, IHC of placental tissues showed a higher expression of NGAL in the fetal surfaces and upper portion of the placenta parenchyma of tobacco exposure mothers (Figure 1A,C) compared to those of No TE (Figure 1B,D) mothers. The IHC for the BPD TE group (Figure 1A) showed higher expression of NGAL as compared to the BPD No TE group (Figure 1B). Regardless of BPD status, NGAL was highly expressed in the TE groups (BPD TE and No BPD TE) compared to the No TE group (BPD No TE and No BPD No TE). Additionally, NGAL intensity staining scores were higher in the chorionic plate and subchorionic space of placentas from tobacco exposure mothers, regardless of BPD status, though these differences did not reach statistical significance (Figure 1E,G; *p* = 0.065 and *p* = 0.091, respectively).

**Figure 2:**
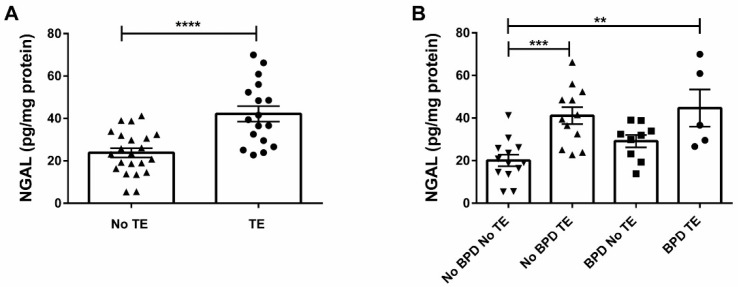
ELISA for NGAL within placental tissue comparing (**A**) TE status and further comparing (**B**) TE and BPD status. (**A**) Compares TE group (n = 17) and No TE group (n = 22)—the NGAL is significantly higher in the TE group compared to No TE group (**** *p* < 0.0001). (**B**) Further subgroup analysis based on BPD status had significantly higher NGAL in the BPD TE group compared to No BPD No TE group (** *p* < 0.01). BPD No TE group also had significantly higher levels of NGAL as compared to No BPD No TE infants (*** p < 0.001). ELISA—enzyme-linked immunosorbent assay, NGAL—neutrophil gelatinase-associated lipocalin, BPD—bronchopulmonary dysplasia, and TE—tobacco exposure.

To confirm these histological findings, NGAL ELISA was performed in each of the four subgroups. As shown in Figure 2A, NGAL levels were significantly higher in the placentas of tobacco exposure compared to No TE mothers (*p* < 0.0001). Further subgroup analysis based on BPD outcomes showed that NGAL levels were significantly higher in infants of the BPD TE group compared to No BPD No TE infants (Figure 2B, *p* < 0.01). Notably, BPD No TE group also had significantly higher levels of NGAL as compared to No BPD No TE infants (Figure 2B, *p* < 0.001). Altogether, these data suggest that tobacco exposure during pregnancy is associated with increased neutrophil activation/infiltration in the placenta, and levels of neutrophil activation/infiltration are increased further still in the placentas of tobacco exposure infants developing BPD.

**Figure A2:**
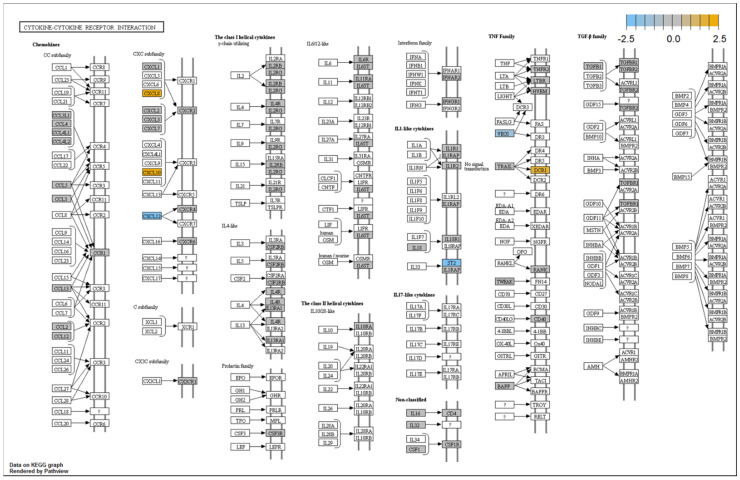
NanoString nSolver^®^ pathway analysis indicating upregulation (yellow) and downregulation (blue) of genes encoding interactions between cytokines and cytokine receptors in BPD TE placentas compared to No BPD No TE.

Next, the immune placental transcriptome from a subset of infants from all four subgroups was profiled using the NanoString nCounter™ Immunology Panel. Comparing BPD TE to No BPD No TE, 22 genes were significantly differentially expressed (Table 3) out of a total of 594 genes of potential interest (Table A3). Notably, transcript levels for the chemokines IL8 and CXCL10, the inflammatory molecules SA100A8/9, and the receptor CD44 were significantly upregulated in BPD TE compared to No BPD No TE infants (Table 3; *p* < 0.05), influencing cell signaling and inflammatory cytokine pathways (e.g., Figure A2). No other significant differences were found between the groups. We further compared the subgroups based on the neonatal outcome of BPD. Similarly, gene expression for CXCL8, CXCL10 were upregulated in the TE BPD group compared to no TE no BPD group.

## 4. Discussion

Bronchopulmonary dysplasia, a disease primarily affecting preterm infants, can be a challenge to manage both acutely and in the long term, as there are many persistent complications affecting patients and their families [24,25]. In this study, we sought to investigate whether tobacco exposure during pregnancy is a risk factor for developing BPD. Specifically, we questioned whether neutrophil activation/infiltration occurs in the placentas of tobacco exposure mothers and if this infiltration of neutrophils to the placenta is associated with the development of BPD or death, as a composite outcome, in preterm infants.

NGAL, neutrophil gelatinase-associated lipocalin, is a 25 kDa lipocalin originally purified from activated human neutrophils. This molecule is now known to be secreted by a variety of immune cells, hepatocytes, adipocytes, and renal tubular cells [26]. In the placenta, NGAL staining has been associated with inflammation and intra-amniotic infections [26]. NGAL levels in the plasma have also been associated with the development of BPD in preterm infants [14]. In this study, we showed for the first time that NGAL staining and NGAL protein levels are higher in the placentas of tobacco exposure mothers compared to those of No tobacco exposure mothers. Using IHC, NGAL staining was specifically high in the amniochorionic membrane and intervillous space, suggesting the presence of neutrophil activation on both the maternal and fetal surfaces. Levels of NGAL measured by ELISA in placenta homogenates were higher in BPD tabacco exposure infants compared to No BPD tobacco exposure infants. Notably, we found no difference in pathologically diagnosed chorioamnionitis or funisitis between the BPD and No BPD groups, suggesting that the observed elevated NGAL levels could be secondary to maternal tobacco exposure.

The potential physiological mechanisms associating maternal tobacco exposure with increased placental NGAL are currently unknown. However, it is reasonable to assume that tobacco exposure during pregnancy results in increased inflammation and immune cell activation, both systemically and at the placenta [27]. Immune cell activation would result in the release of inflammatory cytokines and chemotactic factors [28], potentially affecting the maturation of the fetal lungs. Previous studies have confirmed an association of elevated levels of pro-inflammatory cytokines (interleukin 6 [IL-6], tumor necrosis factor-alpha [TNF-α], IL-1β, and IL-8) in amniotic fluid 5 days preceding delivery with the development of BPD, suggesting that the mechanism responsible for BPD may begin before birth [29].

To determine if tobacco exposure is associated with increased inflammation in the placenta, we profiled the placental tissues as from tobacco exposure and no tobacco exposure mothers using the nCounter^®^ Immunology NanoString Panel, which includes over 500 immunology genes involved with activation of the inflammatory cascade, including neutrophils, natural killer cell, B cell, and T cell activation, as well as various genes responsible for complement activation. Notably, IL8 and CXCL10 mRNA were significantly upregulated in tobacco exposure compared to no tobacco exposure placenta. Both genes encode chemokines known to recruit immune cells, including neutrophils, and are associated with inflammation in the placenta [28,30]. Additionally, the SA100A8 and SA100A9 genes, upregulated in tobacco exposure placentas, encode inflammatory proteins previously shown to play a role in pregnancy loss and other complications, such as preeclampsia [31]. These expression differences further support our suggestion that maternal tobacco exposure is associated with placental inflammation, at least at the transcript level.

Surprisingly, we found no association between maternal tobacco exposure and the incidence of BPD in preterm infants born <32 weeks gestation. This lack of association could be due to the small sample size, as well as a multitude of factors known to be involved in the pathogenesis of BPD [24]. Though a previous study showed a potential association of BPD with maternal tobacco exposure, the majority of the literature indicates that maternal smoking during pregnancy is not an independent risk factor for BPD development, after controlling for additional variables [6,8,32,33]. With the exception of antepartum hemorrhage incidence, which was significantly higher in the composite outcome group compared to the No BPD group (46.7% vs. 4.2%; *p* = 0.003), we found no difference in known risk factors for BPD, including maternal hypertension, PPROM, and chorioamnionitis [8,9,10,11,12]. In line with other studies [7], infants with the composite outcome of BPD or death had a lower gestational age and birth weight compared to infants in the No BPD group. Composite outcome infants also required more medical interventions, such as intubation after birth, medical management of PDA, and development of threshold ROP.

Our pilot study is subject to several limitations. First, maternal tobacco exposure status was based on a self-reported questionnaire rather than biochemical measurement, such as levels of cotinine, a nicotine metabolite. We previously showed that serum cotinine levels were significantly higher in cord blood of self-reported smokers than in cord blood of non-smokers, suggesting that self-reporting smoking status could be adequate in our patient population [21]. Secondly, we did not account for the amount of tobacco exposure (e.g., number of cigarettes smoked per day, or passive versus active smoking) in our results. It is possible that active smoking has a stronger association with placental pathology than passive tobacco exposure. Third, due to the small sample size, we focused on the clinically relevant outcome of moderate to severe BPD and did not adjust for the multiple confounding variables that contribute to the development of BPD. Lastly, our focus in this study was primarily on neutrophil activation. We did not evaluate the effect of tobacco exposure on activation or placental infiltration of other leukocytes.

Our studies provide direct evidence that maternal tobacco exposure leads to neutrophil infiltration into the placenta. One possible implication of this observation is an increased inflammatory environment which could amplify other risk factors, chorioamnionitis, preeclampsia, high oxygen or mechanical ventilation, resulting in the development of BPD [16]. Additional studies need to be carried out focusing on other leukocytes present in the placenta and the cytokines the neonate is exposed to that could contribute to inflammatory injury in the developing lungs. Further, an additional larger study should be carried out to determine if an increase neutrophil infiltration into the placenta due to tobacco exposure is predictive of BPD.

## 5. Conclusions

In conclusion, our studies provide direct evidence that maternal tobacco exposure leads to neutrophil infiltration into the placenta. One possible implication of this observation is an increased inflammatory environment which could amplify other risk factors, chorioamnionitis, preeclampsia, high oxygen or mechanical ventilation, resulting in the development of BPD [16]. Additional studies need to be carried out focusing on other leukocytes present in the placenta and the cytokines the neonate is exposed to that could contribute to inflammatory injury in the developing lungs. Further, an additional larger study should be carried out to determine if an increase neutrophil infiltration into the placenta due to tobacco exposure is predictive of BPD.

## Figures and Tables

**Table 1:** Maternal and Neonatal Demographic Data by Composite Outcome.

	Tobacco Exposure			
	No	Yes		
	(n = 22)	(n = 17)	Total (n = 39)	*p* Value
Birth weight, g (SD)	1.141 (458)	1.125 (464)	1.134 (454)	>0.9
Birth length, cm (SD)	36.3 (4.9)	36.6 (4.6)	36.5 (4.7)	0.9
Head circumference, cm (SD)	25.56 (2.87)	25.48 (3.25)	25.53 (3.00)	>0.9
Gestational age, wk (SD)	28.76 (2.64)	28.40 (2.68)	28.60 (2.63)	0.5
Maternal age, yr (SD)	25.7 (4.9)	29.8 (6.6)	27.5 (6.0)	0.048
Sex				0.4
F	17 (77%)	10 (59%)	27 (69%)	
M	5(23%)	7(41%)	12(31%)	
Maternal ethnicity				0.4
Black	5 (23%)	3 (18%)	8 (21%)	
Black, Native American	0 (0%)	1 (5.9%)	1 (2.6%)	
Hispanic	6 (27%)	2 (12%)	8 (21%)	
Latino, White	1 (4.5%)	0 (0.0%)	1 (2.6%)	
Native American	1 (4.5%)	3 (18%)	4 (10%)	
White	9 (41%)	7 (41%)	16 (41%)	
White, Native American	0 (0%)	1 (5.9%)	1 (2.6%)	
Antenatal steroid, yes	9 (41%)	9 (53%)	18 (46%)	0.4
Mode of delivery, C-Section	11 (50%)	10 (59%)	21 (54%)	0.8
Intubated in delivery room, yes	9 (41%)	9 (53%)	18 (46%)	0.7
Intubated in NICU, yes	7 (32%)	4 (24%)	11 (28%)	0.7
PDA medical treatment, yes	9 (41%)	2 (12%)	11 (28%)	0.073
PDA surgical treatment, yes	1 (4.5%)	0 (0%)	1 (2.6%)	>0.9
IVH grade 3 or 4, yes	2 (9.1%)	1 (5.9%)	3 (7.7%)	>0.9
Threshold ROP, yes	3 (14%)	1 (5.9%)	4 (10%)	0.6
IUGR <10th percentile, yes	3 (14%)	0 (0%)	3 (7.7%)	0.2
Death or BPD, yes	15 (38%)	9 (41%)	6 (35%)	>0.9

All data are presented as the mean (standard deviation) or n (%). Statistical tests performed: Wilcoxon rank-sum test; chi-square test of independence; Fisher’s exact test. *BPD*—bronchopulmonary dysplasia, *NICU*—neonatal intensive care unit, *PDA*—patent ductus arteriosus, *IVH*—intraventricular hemorrhage, *ROP*—retinopathy of prematurity, and *IUGR*—intrauterine growth restriction.

**Table 2:** Maternal Complications and Tobacco Exposure by Composite Outcome.

	Tobacco Exposure			
	No	Yes		
	(n = 22)	(n = 17)	Total (n = 39)	*p* Value
Maternal diabetes, yes	3 (14%)	2 (12%)	5 (13%)	>0.9
Maternal hypertension, yes	3 (14%)	2 (12%)	5 (13%)	>0.9
Prolonged rupture of membranes (>18h), yes	4 (18%)	3 (18%)	7 (18%)	>0.9
Chorioamnionitis, yes	10 (45%)	10 (59%)	20 (51%)	0.6
Antepartum hemorrhage, yes	4 (18%)	4 (24%)	8 (21%)	0.7
Marijuana use, yes	1 (4.5%)	1 (5.9%)	2 (5.1%)	0.4
Illicit drugs, yes	0 (0%)	2 (12%)	2 (5.1%)	0.4

All data are presented as n (%). Statistical tests performed: Fisher’s exact test; chi-square test of independence.

**Table 3:** Significantly Differential Gene Expressions (TE vs. No TE).

Gene	Annotation	Log2-Fold Change	SE	*p*	Tentative Function
IL8	Interleukin 8	4.77	0.898	0.00034	Neutrophil Chemotaxis
S100A9	S100 Calcium-Binding Protein A9	1.72	0.339	0.000477	Leukocyte Activation
S100A8	S100 Calcium-Binding Protein A8	3.33	0.912	0.00447	Leukocyte Activation
IL1RL1	Interleukin 1 Receptor Like 1	−3.41	1.1	0.0115	IL-33 Receptor/Inflammatory Signaling
CXCL10	C-X-C Motif Chemokine Ligand 10	3.06	1.09	0.0187	Peripheral Immune Cell Activation
CD44	CD44 Molecule	1.7	0.614	0.02	Cell–Cell Signaling
TNFRSF10C	TNF Receptor Superfamily Member 10c	1.53	0.562	0.0212	Anti-Apoptosis
PLAUR	Plasminogen Activator, Urokinase Receptor	1.78	0.676	0.0251	Plasminogen Activation/Extracellular Matrix Degradation
IRF7	Interferon Regulatory Factor 7	1.08	0.415	0.0261	Anti-viral Immune Response
MALT1	MALT1 Paracaspase	−0.572	0.22	0.0263	NF-κB Activation
LILRB3	Leukocyte Immunoglobulin-Like Receptor B3	2.04	0.795	0.0281	Anti-B Cell Activation
HLA-DRB1	Major Histocompatibility Complex, Class II, DR Beta 1	2.74	1.14	0.0374	Antigen Presentation
HLA-DRB3	Major Histocompatibility Complex, Class II, DR Beta 3	1.78	0.746	0.0384	Antigen Presentation
HFE	Homeostatic Iron Regulator	−1.25	0.529	0.0394	Regulates Iron Absorption
TNFSF15	TNF Superfamily Member 15	−1.43	0.604	0.0397	Endothelial Inflammatory Signaling
CD99	CD99 Molecule	1.12	0.475	0.0406	Leukocyte Migration
PTPRC	Protein Tyrosine Phosphatase Receptor Type C	1.94	0.847	0.045	T Cell Activation
PTAFR	Platelet-Activating Factor Receptor	−2	0.881	0.0466	Receptor for Inflammatory PAF
ZBTB16	Zinc Finger- and BTB Domain-Containing 16	−2.01	0.888	0.0469	Transcription Repression/Myeloid Maturation
PLA2G2A	Phospholipase A2 Group IIA	−2.25	0.997	0.0479	Phospholipid Metabolism

*BPD*—bronchopulmonary dysplasia, *TE*—tobacco exposure, and *SE*—standard error.

## Data Availability

Data is available on request.

## Appendix A

**Table A1:** Maternal and Neonatal Demographic Data by Composite Outcome.

Maternal and Neonatal Demographic Data				
	No BPD or Death	BPD or Death		
	(n = 22)	(n = 15)	Total (n = 39)	*p* Value
Birth weight (g)	1397.7 ± 355.1	711.7 ± 207.9	1133.8 ± 454.5	<0.001
Birth length (cm)	39.4 ± 2.9	31.7 ± 2.6	36.5 ± 4.7	<0.001
Head circumference (cm)	27.4 ± 1.8	22.5 ± 1.7	25.5 ± 3.0	<0.001
Gestational age (wk)	29.8 ± 1.4	25.5 ± 1.9	28.2 ± 2.6	<0.001
Maternal age (yr)	27.1 ± 5.6	28.2 ± 6.7	27.5 ± 6.0	0.643
Sex				0.083
F	14 (58.3)	13 (86.7)	27 (69.2)	
M	10 (41.7)	2 (13.3)	12 (30.8)	
Maternal ethnicity				0.233
Black	3 (12.5%)	5 (33.3%)	8 (20.5%)	
Black, Native American	0 (0%)	1 (6.7%)	1 (2.6%)	
Hispanic	7 (29.2%)	1 (6.7%)	8 (20.5%)	
Latino, White	1 (4.2%)	0 (0.0%)	1 (2.6%)	
Native American	3 (12.5%)	1 (6.7%)	4 (10.3%)	
White	9 (37.5%)	7 (46.7%)	16(41.0%)	
White, Native American	1 (4.2%)	0 (0.0%)	1 (2.6%)	
Antenatal steroid exposure	23 (95.8)	15 (100)	38 (97.4)	1.000
Mode of delivery, C-Section	14 (58.3)	7 (46.7)	21 (53.8)	0.477
Intubated in delivery room	6 (25)	12 (80)	18 (46.2)	0.001
Intubated in NICU	2 (8.3)	9 (60.0)	11 (28.2)	<0.001
PDA medical treatment	3 (12.5)	8 (53.3)	11 (28.2)	0.010
PDA surgical treatment	0 (0)	1 (6.7)	1 (2.6)	0.385
IVH grade 3 or 4	1 (4.2)	2 (13.3)	3 (7.7)	0.547
Threshold ROP, yes	0 (0)	4 (26.7)	4 (10.3)	0.017
IUGR <10th percentile, yes	1 (4.2)	2 (13.3)	3 (7.7)	0.547

All data are presented as the mean ± standard deviation or *n* (%). *BPD*—bronchopulmonary dysplasia, *NICU*—neonatal intensive care unit, *PDA*—patent ductus arteriosus, *IVH*—intraventricular hemorrhage, *ROP*—retinopathy of prematurity, and *IUGR*—intrauterine growth restriction.

**Table A2:** Maternal Complications and Tobacco Exposure by Composite Outcome.

	No BPD or Death	BPD or Death		
	(n = 24)	(n = 15)	Total (n = 39)	*p* Value
Maternal diabetes	4 (16.7)	1 (6.7)	5 (12.8)	0.634
Maternal hypertension	3 (12.5)	2 (13.3)	5 (12.8)	1.000
Prolonged rupture of membranes (>18h)	3 (12.5)	4 (26.7)	7 (17.9)	0.396
Histological chorioamnionitis	12 (50)	8 (53.3)	20 (51.3)	0.839
Antepartum hemorrhage	1 (4.2)	7 (46.7)	8 (20.5)	0.003
Maternal TE	11 (45.8)	6 (40)	17 (43.6)	0.721
Maternal active smoking	9 (37.5)	3 (20)	12 (30.8)	0.305
Maternal passive smoke exposure	11 (45.8)	7 (46.7)	18 (46.2)	0.959
Illicit drugs, yes	1 (4.21)	1 (6.7)	2 (5.1)	0.765

All data are presented as *n* (%). *BPD*—bronchopulmonary dysplasia, *PPROM*—premature prolonged rupture of membranes, and *TE*—tobacco exposure.

**Table A3:** NanoString Gene Expressions (BPD TE vs. No BPD No TE).

Gene	Log2-Fold Change	SE	Lower Confidence Limit (log2)	Upper Confidence Limit (log2)	*p*	Tentative Function
IL8	4.77	0.898	3.01	6.54	0.00034	Cell Activation
S100A9	1.72	0.339	1.06	2.38	0.000477	Cell–Cell Signaling
S100A8	3.33	0.912	1.54	5.12	0.00447	Defense Response
IL1RL1	−3.41	1.1	−5.57	−1.24	0.0115	Receptor Signaling Protein Activity
CXCL10	3.06	1.09	0.922	5.21	0.0187	Behavior
CD44	1.7	0.614	0.493	2.9	0.02	Cell–Cell Signaling
TNFRSF10C	1.53	0.562	0.433	2.64	0.0212	Integral to Membrane
PLAUR	1.78	0.676	0.454	3.11	0.0251	Behavior
IRF7	1.08	0.415	0.269	1.9	0.0261	Biopolymer Metabolic Process
MALT1	−0.572	0.22	−1	−0.141	0.0263	Cell Development
LILRB3	2.04	0.795	0.481	3.6	0.0281	Cell Surface Receptor-Linked Signal Transduction
HLA-DRB1	2.74	1.14	0.502	4.99	0.0374	Antigen Presentation
HLA-DRB3	1.78	0.746	0.316	3.24	0.0384	Immune Response
HFE	−1.25	0.529	−2.29	−0.216	0.0394	Cytoplasm
TNFSF15	−1.43	0.604	−2.61	−0.244	0.0397	Cell Development
CD99	1.12	0.475	0.186	2.05	0.0406	Cytoplasm
PTPRC	1.94	0.847	0.28	3.6	0.045	Integral to Membrane
PTAFR	−2	0.881	−3.73	−0.273	0.0466	Behavior
ZBTB16	−2.01	0.888	−3.75	−0.272	0.0469	Intracellular Organelle Part
PLA2G2A	−2.25	0.997	−4.2	−0.292	0.0479	Cytoplasm
CXCL12	−2.06	0.92	−3.86	−0.256	0.0492	Behavior
HRAS	−1.39	0.621	−2.6	−0.169	0.0497	Anatomical Structure Development
SELL	1.66	0.757	0.177	3.15	0.053	Integral to Membrane
PSMB9	1.13	0.578	−0.00586	2.26	0.0798	Antigen Presentation
NT5E	0.917	0.482	−0.0275	1.86	0.0862	Biopolymer Metabolic Process
CCL3	1.66	0.885	−0.0776	3.39	0.0907	Cell Fraction
CD83	1.64	0.884	−0.0944	3.37	0.0935	Defense Response
NFKBIA	0.72	0.398	−0.0604	1.5	0.101	Apoptosis
HLA-DRA	1.16	0.648	−0.114	2.43	0.105	Cytoplasm
CLEC4A	1.27	0.71	−0.125	2.66	0.105	Cell Surface Receptor-Linked Signal Transduction
HLA-C	1.22	0.686	−0.124	2.57	0.106	Antigen Presentation
CXCL1	1.66	0.942	−0.184	3.51	0.108	Behavior
BCL3	1.25	0.717	−0.158	2.65	0.112	Cytoplasm
ITGAX	1.77	1.03	−0.243	3.79	0.115	Anatomical Structure Morphogenesis
HLA-DMA	0.667	0.396	−0.11	1.44	0.123	Antigen Presentation
TRAF5	−1.05	0.631	−2.29	0.183	0.126	IκB Kinase NFκB Cascade
HLA-A	0.723	0.436	−0.131	1.58	0.128	Antigen Presentation
GATA3	−1.13	0.679	−2.46	0.204	0.128	Anatomical Structure Morphogenesis
CD74	1.29	0.78	−0.237	2.82	0.129	Biosynthetic Process
LILRB2	1.22	0.737	−0.228	2.66	0.13	Cell–Cell Signaling
BST1	1.07	0.649	−0.205	2.34	0.131	Humoral Immune Response
LTB4R2	−0.977	0.594	−2.14	0.187	0.131	Behavior
RARRES3	−1.23	0.764	−2.73	0.268	0.139	Cell Proliferation
TNFSF13B	0.903	0.566	−0.207	2.01	0.142	Cell Fraction
XBP1	1.34	0.84	−0.307	2.99	0.142	DNA Binding
CD24	1.22	0.778	−0.299	2.75	0.146	Cell Surface
NFKB2	0.877	0.56	−0.221	1.98	0.148	Biopolymer Metabolic Process
ITGAE	−0.775	0.505	−1.77	0.215	0.156	Integral to Membrane
VCAM1	−1.04	0.69	−2.39	0.312	0.162	Leukocyte Adhesion
PSMD7	0.342	0.229	−0.106	0.79	0.165	Macromolecular Complex
ATG12	−1.03	0.691	−2.38	0.328	0.168	Apoptosis
CXCL2	1.32	0.896	−0.434	3.08	0.171	Behavior
MAPK11	0.721	0.497	−0.254	1.7	0.178	Intracellular Signaling Cascade
IL11RA	−0.872	0.603	−2.05	0.311	0.179	Integral to Membrane
TAL1	−1.01	0.702	−2.39	0.364	0.18	Cell Proliferation
PPBP	1.06	0.736	−0.382	2.5	0.18	Establishment of Localization
TNFRSF14	−0.854	0.601	−2.03	0.324	0.186	Cell Surface Receptor-Linked Signal Transduction
ITGAM	0.932	0.659	−0.359	2.22	0.187	Integral to Membrane
C2	−0.415	0.294	−0.991	0.161	0.188	Defense Response
CD59	0.626	0.446	−0.247	1.5	0.19	Cell Fraction
TGFB1	1.15	0.817	−0.455	2.75	0.191	DNA Metabolic Process
MIF	0.686	0.489	−0.272	1.65	0.191	Biosynthetic Process
FCGR2A	1.97	1.41	−0.789	4.72	0.192	Phagocytosis
BATF3	−0.858	0.62	−2.07	0.358	0.197	Biopolymer Metabolic Process
CCL4	0.946	0.685	−0.397	2.29	0.198	Anatomical Structure Morphogenesis
IFI35	−0.559	0.406	−1.36	0.237	0.199	Nucleus
HLA-DPB1	1.39	1.01	−0.591	3.36	0.199	Multi-Organism Process
FCER1G	0.953	0.699	−0.417	2.32	0.203	Integral to Membrane
FCGR3A/B	2.03	1.49	−0.902	4.95	0.205	Immune Response
HLA-DPA	1.33	1	−0.639	3.3	0.215	Antigen Presentation
SRC	−0.655	0.497	−1.63	0.32	0.217	Cell Surface Receptor-Linked Signal Transduction
IL1R2	0.888	0.681	−0.446	2.22	0.221	Immune Response
CXCR2	0.877	0.675	−0.447	2.2	0.223	Receptor for IL-8
ITGAL	1.15	0.894	−0.598	2.91	0.226	Leukocyte Adhesion
CFD	−0.558	0.433	−1.41	0.29	0.226	Cellular Macromolecule Metabolic Process
SOCS3	0.87	0.681	−0.464	2.2	0.23	Cell Development
IL2RG	0.973	0.763	−0.522	2.47	0.231	Cell Surface
PECAM1	−0.491	0.386	−1.25	0.265	0.232	Membrane
TNFRSF1B	1.34	1.06	−0.729	3.41	0.233	Receptor Activity
CASP8	1.27	1.02	−0.725	3.27	0.241	Cell Development
GBP1	1.41	1.13	−0.81	3.63	0.242	Cell Metabolism
TLR2	0.784	0.644	−0.478	2.05	0.251	Cell Development
CDKN1A	−0.618	0.51	−1.62	0.381	0.253	Cell Development
S1PR1	0.794	0.662	−0.503	2.09	0.258	S1P Receptor
IL18	0.562	0.475	−0.369	1.49	0.264	Anatomical Structure Morphogenesis
TFRC	−0.715	0.632	−1.95	0.523	0.284	Cytoplasm
VTN	−1.19	1.07	−3.29	0.904	0.291	Extracellular Region
GPI	1.29	1.16	−0.987	3.56	0.293	Hemostasis
MR1	−0.763	0.689	−2.11	0.587	0.294	Immune Response
PRKCD	0.638	0.578	−0.495	1.77	0.296	Biopolymer Metabolic Process
BCL10	1.35	1.22	−1.05	3.74	0.296	Cytoplasm
MAPK14	1.55	1.4	−1.21	4.3	0.297	Behavior
ZEB1	−0.667	0.607	−1.86	0.521	0.297	Biopolymer Metabolic Process
EBI3	0.721	0.661	−0.574	2.02	0.301	Biosynthetic Process
PTPN2	1.05	0.969	−0.849	2.95	0.304	Biopolymer Metabolic Process
TNFRSF11A	−0.464	0.43	−1.31	0.379	0.306	Cell–Cell Signaling
IL32	0.781	0.733	−0.655	2.22	0.312	Defense Response
C1QA	1.34	1.27	−1.15	3.84	0.317	Cell–Cell Signaling
CHUK	1.23	1.18	−1.08	3.54	0.322	Anatomical Structure Morphogenesis
AHR	−0.603	0.579	−1.74	0.533	0.323	Biopolymer Metabolic Process
TGFBR2	−0.33	0.323	−0.963	0.304	0.331	Cell Proliferation
IL13RA1	0.217	0.214	−0.202	0.637	0.334	Cell Surface Receptor-Linked Signal Transduction
PDCD1LG2	−0.741	0.732	−2.18	0.694	0.335	Antigen Presentation
ETS1	−0.659	0.666	−1.96	0.647	0.346	Hemopoiesis
FADD	0.531	0.538	−0.524	1.58	0.347	Cell Development
HLA-B	1.45	1.47	−1.43	4.33	0.348	Cell Fraction
MYD88	1.15	1.17	−1.15	3.45	0.352	IκB Kinase NFκB Cascade
CR1	0.723	0.742	−0.731	2.18	0.353	Integral to Membrane
TGFBI	1.51	1.55	−1.53	4.54	0.353	Cell Proliferation
TRAF6	−0.132	0.137	−0.401	0.136	0.358	Biopolymer Metabolic Process
LTBR	1.26	1.33	−1.33	3.86	0.363	IκB Kinase NFκB Cascade
TLR7	−0.604	0.637	−1.85	0.644	0.365	Biosynthetic Process
BCAP31	−0.968	1.02	−2.97	1.03	0.366	Integral to Membrane
CD45R0	0.666	0.707	−0.719	2.05	0.368	Integral to Membrane
PSMC2	1.35	1.45	−1.5	4.19	0.375	Cytoplasm
CUL9	−0.547	0.596	−1.72	0.622	0.381	Microtubule Dynamics
MAP4K4	0.236	0.258	−0.269	0.742	0.381	Biopolymer Metabolic Process
TLR4	1.05	1.15	−1.2	3.3	0.381	Biosynthetic Process
STAT6	1.09	1.2	−1.25	3.44	0.383	DNA Binding
LTF	1.28	1.4	−1.47	4.02	0.384	Endopeptidase Activity
STAT3	0.35	0.385	−0.404	1.1	0.384	Biopolymer Metabolic Process
BCL6	0.734	0.808	−0.849	2.32	0.385	Intracellular Non-Membrane-Bound Organelle
FYN	0.626	0.695	−0.735	1.99	0.389	Behavior
IKBKAP	0.885	0.986	−1.05	2.82	0.39	Cytoplasm
PPARG	0.944	1.06	−1.13	3.01	0.393	Biopolymer Metabolic Process
IFITM1	1.26	1.42	−1.52	4.05	0.395	Cell Proliferation
CD40	−0.655	0.741	−2.11	0.797	0.398	Defense Response
CASP3	1.03	1.17	−1.26	3.33	0.398	Cell Development
TAPBP	0.332	0.383	−0.42	1.08	0.407	Cytoplasm
IFI16	1.09	1.27	−1.4	3.58	0.411	Cell Development
CD45RB	0.708	0.825	−0.91	2.33	0.411	Integral to Membrane
GPR183	−0.755	0.882	−2.48	0.973	0.412	Unknown
TAGAP	−0.671	0.79	−2.22	0.877	0.416	Rho GTPase Activation
ITGB2	0.619	0.732	−0.815	2.05	0.417	Behavior
NFATC2	0.97	1.15	−1.28	3.22	0.418	Biopolymer Metabolic Process
TAP2	0.575	0.682	−0.761	1.91	0.419	Cytoplasm
TBK1	1.12	1.33	−1.49	3.74	0.42	IκB Kinase NFκB Cascade
NCF4	0.51	0.607	−0.681	1.7	0.421	Cytoplasm
PTPN6	0.984	1.17	−1.32	3.29	0.422	Biopolymer Metabolic Process
ILF3	1.15	1.38	−1.55	3.85	0.423	Biopolymer Metabolic Process
CASP2	0.999	1.2	−1.35	3.34	0.423	Apoptosis
CD274	0.345	0.415	−0.468	1.16	0.425	Cell Proliferation
SPP1	−0.645	0.779	−2.17	0.882	0.427	Osteoclast Attachment
FCGR1A/B	0.507	0.613	−0.695	1.71	0.428	Establishment of Localization
RELA	0.375	0.461	−0.528	1.28	0.435	Cell Development
SERPING1	1.26	1.55	−1.78	4.3	0.436	Regulation of Complement
TNFSF10	1.13	1.41	−1.64	3.9	0.441	Cell Development
CSF2RB	0.579	0.732	−0.856	2.01	0.447	Integral to Membrane
NFKBIZ	0.951	1.21	−1.42	3.32	0.45	Activation of IL-6
PTK2	1.17	1.49	−1.76	4.1	0.452	Cell Surface Receptor-Linked Signal Transduction
RUNX1	−0.403	0.52	−1.42	0.617	0.456	Biopolymer Metabolic Process
CTNNB1	1.44	1.86	−2.2	5.08	0.457	Cell Development
FCGR2B	−0.39	0.507	−1.38	0.604	0.46	Immune Response
TNFAIP3	0.511	0.665	−0.792	1.81	0.46	IκB Kinase NFκB Cascade
IL1R1	−1	1.32	−3.58	1.58	0.463	Integral to Membrane
MAPKAPK2	−0.436	0.573	−1.56	0.687	0.464	Biopolymer Metabolic Process
CD209	0.649	0.854	−1.03	2.32	0.465	Cell–Cell Adhesion
MX1	−0.522	0.689	−1.87	0.828	0.466	Apoptosis
PSMB7	0.242	0.321	−0.386	0.871	0.467	Peptide Cleavage
ATG7	0.881	1.17	−1.42	3.18	0.47	Biopolymer Metabolic Process
CTSC	1.31	1.74	−2.11	4.72	0.47	Cytoplasm
IL18R1	−0.535	0.734	−1.97	0.903	0.482	Membrane
CCL5	−0.467	0.648	−1.74	0.803	0.487	Behavior
LILRB4	−0.632	0.877	−2.35	1.09	0.488	Antigen Binding
IL2RB	0.594	0.83	−1.03	2.22	0.491	Cell Development
PSMB5	−0.26	0.364	−0.973	0.453	0.491	Peptide Cleavage
CD46	1.35	1.9	−2.37	5.08	0.492	Integral to Membrane
C1QB	1.2	1.7	−2.12	4.53	0.494	Extracellular Region
C14orf166	1.11	1.58	−1.98	4.2	0.499	Identical Protein Binding
FCGR2A/C	1.08	1.59	−2.03	4.19	0.513	Phagocyte Cell Surface Receptor
LCP2	0.465	0.687	−0.881	1.81	0.514	Cell Surface Receptor-Linked Signal Transduction
EGR1	−0.499	0.746	−1.96	0.962	0.519	Transcriptional Regulation
CD28	−0.393	0.598	−1.56	0.779	0.526	Cell Development
TRAF2	−0.126	0.194	−0.506	0.253	0.528	Macromolecular Complex Assembly
NOD2	0.524	0.806	−1.06	2.1	0.531	Cytoplasm
TLR1	−0.302	0.468	−1.22	0.616	0.534	Biosynthetic Process
SMAD5	0.762	1.19	−1.58	3.1	0.538	Cell Surface Receptor-Linked Signal Transduction
PML	0.387	0.607	−0.803	1.58	0.538	Cell Fraction
CXCR4	0.485	0.764	−1.01	1.98	0.54	Cell Development
IRF1	−0.178	0.283	−0.732	0.376	0.542	Biopolymer Metabolic Process
LGALS3	1.02	1.63	−2.17	4.2	0.545	Carbohydrate Binding
ENTPD1	−0.483	0.777	−2.01	1.04	0.548	Hemostasis
ICOSLG	−0.415	0.67	−1.73	0.898	0.55	Cell Activation
BCL2	0.496	0.801	−1.07	2.06	0.55	Cytoplasm
STAT2	0.804	1.3	−1.74	3.35	0.55	Biopolymer Metabolic Process
CD86	−0.478	0.773	−1.99	1.04	0.55	Cell Proliferation
CD276	0.844	1.38	−1.86	3.54	0.554	Biosynthetic Process
MAF	0.342	0.562	−0.759	1.44	0.556	Biopolymer Metabolic Process
IFIT2	−0.568	0.944	−2.42	1.28	0.56	Innate Immune
CD14	0.893	1.49	−2.02	3.81	0.561	Apoptosis
CD81	1.08	1.8	−2.45	4.61	0.562	Integral to Membrane
ITGA5	−0.459	0.784	−2	1.08	0.571	Integral to Membrane
DPP4	0.884	1.51	−2.08	3.84	0.571	Immune Response
CSF1R	−0.326	0.564	−1.43	0.78	0.577	Cell Proliferation
IRF3	0.227	0.393	−0.544	0.997	0.577	Biopolymer Metabolic Process
JAK2	0.736	1.29	−1.79	3.26	0.581	Anatomical Structure Development
ICAM1	0.383	0.673	−0.936	1.7	0.581	Integral to Membrane
FKBP5	−0.396	0.697	−1.76	0.969	0.582	Cellular Macromolecule Metabolic Process
TNFSF12	0.344	0.608	−0.847	1.54	0.583	Cell Development
MRC1	0.841	1.5	−2.09	3.77	0.587	Carbohydrate Binding
SYK	0.343	0.612	−0.856	1.54	0.587	Anatomical Structure Morphogenesis
RELB	0.316	0.564	−0.79	1.42	0.588	DNA Binding
IRF5	0.369	0.666	−0.935	1.67	0.591	Immune Transcription Factor
IRF8	0.368	0.681	−0.967	1.7	0.601	Biopolymer Metabolic Process
SKI	0.684	1.27	−1.8	3.17	0.602	Repressor of TGF-β
C3	0.517	0.96	−1.37	2.4	0.602	Cell Surface Receptor-Linked Signal Transduction
PTGS2	−0.508	0.945	−2.36	1.34	0.603	Cytoplasm
LITAF	0.354	0.659	−0.938	1.65	0.603	Biopolymer Metabolic Process
BLNK	−0.312	0.586	−1.46	0.836	0.606	Anatomical Structure Development
IFNAR2	0.174	0.326	−0.466	0.813	0.607	Cell Surface Receptor-Linked Signal Transduction
MUC1	0.935	1.79	−2.57	4.44	0.613	Integral to Membrane
NOD1	−0.62	1.2	−2.97	1.73	0.616	Biosynthetic Process
PDCD2	0.275	0.534	−0.772	1.32	0.618	Apoptosis
CD82	−0.326	0.638	−1.58	0.925	0.62	Integral to Membrane
NFIL3	0.593	1.18	−1.72	2.91	0.626	Biopolymer Metabolic Process
TCF7	0.363	0.736	−1.08	1.81	0.633	Biopolymer Metabolic Process
ATG10	−0.576	1.18	−2.88	1.73	0.635	Autophagocytosis
LILRB1	0.405	0.838	−1.24	2.05	0.639	Integral to Membrane
FCGRT	0.757	1.58	−2.33	3.85	0.641	Immune Response
ICAM3	−0.32	0.673	−1.64	0.999	0.645	Integral to Membrane
STAT1	0.33	0.695	−1.03	1.69	0.645	Biopolymer Metabolic Process
IL6ST	0.905	1.91	−2.84	4.65	0.645	Cell Surface Receptor-Linked Signal Transduction
PDGFRB	−0.863	1.85	−4.48	2.76	0.65	Phosphotransferase Activity Alcohol Group as Acceptor
CSF1	0.335	0.722	−1.08	1.75	0.653	Cell Proliferation
IL1RAP	0.358	0.782	−1.17	1.89	0.657	Cellular Component Assembly
MCL1	0.13	0.288	−0.434	0.695	0.66	Cell Development
NOTCH2	0.332	0.744	−1.13	1.79	0.665	Cell Development
IFNGR1	0.755	1.71	−2.59	4.1	0.668	Integral to Membrane
NFATC1	−0.346	0.782	−1.88	1.19	0.668	Biopolymer Metabolic Process
SMAD3	−0.262	0.594	−1.43	0.902	0.668	Macromolecular Complex
STAT4	−0.358	0.812	−1.95	1.23	0.669	DNA Binding
C1S	−0.607	1.39	−3.33	2.11	0.671	Endopeptidase Activity
CX3CR1	0.364	0.859	−1.32	2.05	0.681	Behavior
LTB4R	0.322	0.77	−1.19	1.83	0.685	Cell Surface Receptor-Linked Signal Transduction
ARHGDIB	0.307	0.741	−1.15	1.76	0.687	Cytoplasm
JAK3	0.239	0.59	−0.917	1.4	0.693	Biopolymer Metabolic Process
TLR8	0.301	0.749	−1.17	1.77	0.696	Biosynthetic Process
RAF1	0.533	1.33	−2.07	3.14	0.697	Biopolymer Metabolic Process
CSF3R	0.22	0.556	−0.87	1.31	0.701	Defense Response
SIGIRR	0.272	0.693	−1.09	1.63	0.703	Membrane
ATG16L1	−0.436	1.13	−2.66	1.79	0.709	Autophagy
SOCS1	−0.553	1.46	−3.41	2.3	0.712	Biopolymer Metabolic Process
POU2F2	0.26	0.694	−1.1	1.62	0.715	Biopolymer Metabolic Process
HLA-DMB	−0.311	0.839	−1.96	1.33	0.719	Antigen Presentation
MAP4K2	−0.52	1.41	−3.28	2.24	0.72	Biopolymer Metabolic Process
IFIH1	0.186	0.509	−0.812	1.18	0.722	B-Cell Differentiation
TGFBR1	0.5	1.38	−2.21	3.21	0.726	Biopolymer Metabolic Process
B2M	−0.212	0.59	−1.37	0.944	0.726	Antimicrobial Protein
STAT5A	−0.286	0.804	−1.86	1.29	0.729	DNA Binding
IGF2R	0.436	1.27	−2.05	2.92	0.738	Cytoplasm
CD34	−0.175	0.516	−1.19	0.836	0.741	Carbohydrate Binding
ITGB1	−0.191	0.563	−1.29	0.912	0.741	Cell–Cell Adhesion
TLR3	−0.329	0.974	−2.24	1.58	0.742	Biosynthetic Process
CCL13	−0.311	0.926	−2.13	1.5	0.744	Behavior
LAMP3	0.248	0.739	−1.2	1.7	0.744	Cell Proliferation
CCBP2	−0.174	0.522	−1.2	0.85	0.746	Behavior
IDO1	0.261	0.787	−1.28	1.8	0.747	Tryptophan Catabolism
MME	0.244	0.739	−1.2	1.69	0.748	Cell–Cell Signaling
MSR1	0.487	1.48	−2.4	3.38	0.748	Establishment of Localization
C7	−0.292	0.908	−2.07	1.49	0.754	Integral to Membrane
CD36	0.603	1.88	−3.09	4.3	0.755	Cell Fraction
IL16	−0.147	0.462	−1.05	0.759	0.757	Extracellular Region
CISH	−0.232	0.733	−1.67	1.21	0.759	Suppressor of Cytokine Signaling
CCL2	0.256	0.827	−1.37	1.88	0.763	Biopolymer Metabolic Process
CD163	−0.265	0.886	−2	1.47	0.771	Integral to Membrane
STAT5B	0.502	1.69	−2.8	3.81	0.772	DNA Binding
SLC2A1	−0.123	0.419	−0.944	0.698	0.776	Cell Fraction
IRAK4	−0.372	1.29	−2.91	2.16	0.779	Activates NFκB
DUSP4	0.203	0.708	−1.18	1.59	0.78	Biopolymer Metabolic Process
CEBPB	−0.158	0.553	−1.24	0.925	0.78	Biopolymer Metabolic Process
ITGA4	0.213	0.748	−1.25	1.68	0.782	Identical Protein Binding
CTSS	0.22	0.819	−1.39	1.83	0.794	Cellular Macromolecule Metabolic Process
IKZF2	−0.362	1.35	−3.01	2.28	0.794	Lymphocyte Development
LY96	0.189	0.711	−1.2	1.58	0.796	Cell Surface Receptor-Linked Signal Transduction
CLEC7A	0.214	0.812	−1.38	1.81	0.797	Cell Activation
HAVCR2	−0.207	0.787	−1.75	1.34	0.798	Th1 Surface Protein
ICAM2	−0.388	1.48	−3.29	2.51	0.798	Integral to Membrane
PSMB8	0.354	1.35	−2.3	3	0.799	Antigen Presentation
C1R	−0.243	0.945	−2.09	1.61	0.802	Endopeptidase Activity
ABL1	−0.375	1.48	−3.27	2.52	0.805	Biopolymer Metabolic Process
TLR5	−0.117	0.466	−1.03	0.796	0.807	Innate Immunity
BST2	−0.406	1.64	−3.63	2.81	0.81	IκB Kinase NFκB Cascade
IL6R	0.159	0.653	−1.12	1.44	0.813	Cell Surface Receptor-Linked Signal Transduction
TMEM173	0.139	0.642	−1.12	1.4	0.833	Innate Immunity
IL10RA	−0.18	0.835	−1.82	1.46	0.833	Interleukin Binding
CDH5	−0.401	1.9	−4.13	3.33	0.837	Cell–Cell Adhesion
PSMB10	0.273	1.31	−2.3	2.85	0.839	Humoral Immune Response
NFATC3	0.257	1.24	−2.17	2.68	0.84	Biopolymer Metabolic Process
BCL2L11	0.108	0.551	−0.972	1.19	0.849	Apoptosis
EDNRB	−0.308	1.62	−3.49	2.87	0.853	Integral to Membrane
TICAM1	0.119	0.632	−1.12	1.36	0.854	IκB Kinase NFκB Cascade
JAK1	−0.286	1.56	−3.35	2.77	0.858	Interferon Signal Transduction
MAP4K1	0.115	0.632	−1.12	1.35	0.859	Biopolymer Metabolic Process
C1QBP	−0.0783	0.452	−0.964	0.807	0.866	Immune Response
CD97	0.302	1.76	−3.15	3.75	0.867	Cell–Cell Signaling
CMKLR1	−0.271	1.6	−3.41	2.86	0.869	Behavior
ABCB1	−0.109	0.655	−1.39	1.17	0.871	Cell Fraction
ITGA6	−0.0569	0.384	−0.81	0.696	0.885	Cellular Component Assembly
CFI	−0.123	0.832	−1.75	1.51	0.885	Endopeptidase Activity
TOLLIP	−0.227	1.55	−3.27	2.81	0.886	Cell–Cell Signaling
CFH	−0.144	1.02	−2.14	1.85	0.89	Extracellular Region
NFKB1	−0.177	1.26	−2.65	2.3	0.891	Biopolymer Metabolic Process
FN1	−0.0987	0.729	−1.53	1.33	0.895	Cytoplasm
IRAK3	0.114	0.846	−1.54	1.77	0.896	Biopolymer Metabolic Process
CXCR6	0.0995	0.75	−1.37	1.57	0.897	Cell Surface Receptor-Linked Signal Transduction
TCF4	0.0836	0.707	−1.3	1.47	0.908	Biopolymer Metabolic Process
LILRB5	0.0831	0.747	−1.38	1.55	0.914	Cell Surface Receptor-Linked Signal Transduction
IKBKB	0.0595	0.55	−1.02	1.14	0.916	Activates NFκB
IKBKE	0.0559	0.578	−1.08	1.19	0.925	Biopolymer Metabolic Process
CD19	0.126	1.36	−2.54	2.79	0.928	Cell Surface Receptor-Linked Signal Transduction
IRAK1	−0.119	1.39	−2.85	2.61	0.934	Cellular Component Assembly
UBE2L3	−0.112	1.49	−3.03	2.8	0.942	Biopolymer Metabolic Process
CD53	0.126	1.73	−3.26	3.52	0.943	Membrane
TRAF4	−0.1	1.41	−2.87	2.67	0.945	DNA Binding
THY1	−0.0637	0.979	−1.98	1.86	0.949	Cell Surface
ATG5	−0.108	1.75	−3.53	3.31	0.952	Cytoplasm
CEACAM1	0.0489	0.824	−1.57	1.66	0.954	Cell Fraction
CCND3	0.0844	1.55	−2.96	3.13	0.958	Biopolymer Metabolic Process
MAPK1	−0.0932	1.94	−3.89	3.7	0.963	Behavior
CD164	−0.0906	1.98	−3.96	3.78	0.964	Cell–Cell Adhesion
NOTCH1	0.03	0.657	−1.26	1.32	0.965	Cell Development
CRADD	−0.0272	0.617	−1.24	1.18	0.966	Apoptosis
TP53	−0.0682	1.76	−3.52	3.38	0.97	Cell Fraction
BAX	0.0666	1.81	−3.47	3.61	0.971	Cytoplasm
CASP1	0.0524	1.43	−2.75	2.86	0.971	Cellular Protein Metabolic Process
IKBKG	−0.0416	1.19	−2.37	2.28	0.973	Cell Development
TYK2	0.0467	1.37	−2.63	2.73	0.973	Biopolymer Metabolic Process
CLEC4E	−0.0351	1.08	−2.16	2.09	0.975	Carbohydrate Binding
CFB	0.0283	0.901	−1.74	1.79	0.976	Complement Activation
LAIR1	−0.0191	0.739	−1.47	1.43	0.98	Inhibitory Receptor
CD58	−0.0354	1.4	−2.79	2.72	0.98	T Cell Activation
KCNJ2	0.00799	0.384	−0.744	0.76	0.984	Establishment of Localization
PLAU	−0.016	0.788	−1.56	1.53	0.984	Behavior
TRAF3	0.0112	0.57	−1.11	1.13	0.985	Apoptosis
CCR1	0.0271	1.38	−2.68	2.74	0.985	Cell–Cell Signaling
CD9	−0.0138	0.765	−1.51	1.48	0.986	Anatomical Structure Morphogenesis
APP	−0.00668	0.389	−0.769	0.756	0.987	Cell Surface
ARG2	0.0106	0.761	−1.48	1.5	0.989	Cytoplasm
IL4R	0.0159	1.64	−3.21	3.24	0.992	Immune Response
CD4	0.0114	1.48	−2.88	2.91	0.994	Cell Activation
CYBB	0.00269	0.554	−1.08	1.09	0.996	Defense Response
KIT	−4.48 × 10 ^−16^	0.576	−1.13	1.13	1	Phosphotransferase Activity Alcohol Group as Acceptor

## References

[1] Drake P., Driscoll A.K., Mathews T.J. (2018). Cigarette Smoking During Pregnancy.

[2] Spencer K., Cowans N.J. (2013). Accuracy of self-reported smoking status in first trimester aneuploidy screening. Prenat. Diagn..

[3] Kyrklund-Blomberg N.B., Cnattingius S. (1998). Preterm birth and maternal smoking: Risks related to gestational age and onset of delivery. J. Obstet. Gynaecol..

[4] Nicoletti D. (2014). Maternal smoking during pregnancy and birth defects in children: A systematic review with meta-analysis. Cad. Saude Publica.

[5] Hackshaw A., Rodeck C., Boniface S. (2011). Maternal smoking in pregnancy and birth defects: A systematic review based on 173 687 malformed cases and 11.7 million controls. Hum. Reprod. Update.

[6] Antonucci R., Contu P., Porcella A., Atzeni C., Chiappe S. (2004). Intrauterine smoke exposure: A new risk factor for bronchopulmonary dysplasia?. J. Perinat. Med..

[7] Jensen E.A., Schmidt B. (2014). Epidemiology of bronchopulmonary dysplasia. Birth Defects Res. Part A Clin. Mol. Teratol..

[8] Morrow L.A., Wagner B.D., Ingram D.A., Poindexter B.B., Schibler K., Cotten C.M., Dagle J., Sontag M.K., Mourani P.M., Abman S.H. (2017). Antenatal Determinants of Bronchopulmonary Dysplasia and Late Respiratory Disease in Preterm Infants. Am. J. Respir. Crit. Care Med..

[9] Watterberg K.L., Demers L.M., Scott S.M., Murphy S. (1996). Chorioamnionitis and Early Lung Inflammation in Infants in Whom Bronchopulmonary Dysplasia Develops. Pediatrics.

[10] Hansen A.R., Barnés C.M., Folkman J., McElrath T.F. (2010). Maternal Preeclampsia Predicts the Development of Bronchopulmonary Dysplasia. J. Pediatrics.

[11] Eriksson L., Haglund B., Odlind V., Altman M., Ewald U., Kieler H. (2015). Perinatal conditions related to growth restriction and inflammation are associated with an increased risk of bronchopulmonary dysplasia. Acta Paediatr..

[12] Gemmell L., Martin L., Murphy K.E., Modi N., Håkansson S., Reichman B., Lui K., Kusuda S., Sjörs G., Mirea L. (2016). Hypertensive disorders of pregnancy and outcomes of preterm infants of 24 to 28 weeks’ gestation. J. Perinatol..

[13] Xu S., Venge P. (2000). Lipocalins as biochemical markers of disease. Biochim. Biophys. Acta (BBA)-Protein Struct. Mol. Enzymol..

[14] Inoue H., Ohga S., Kusuda T., Kitajima J., Kinjo T., Ochiai M., Takahata Y., Honjo S., Hara T. (2013). Serum neutrophil gelatinase-associated lipocalin as a predictor of the development of bronchopulmonary dysplasia in preterm infants. Early Hum. Dev.

[15] Naeye R.L. (1978). Effects of maternal cigarette smoking on the fetus and placenta. BJOG. Int. J. Obstet..

[16] Jobe A.H. (2016). Mechanisms of lung injury and bronchopulmonary dysplasia. Am. J. Perinatol..

[17] Gonzalez-Luis G.E., Westering-Kroon E.V., Villamor-Martinez E., Huizing M.J., Kilani M.A., Kramer B.W., Villamor E. (2020). Tobacco smoking during pregnancy is associated with increased risk of moderate/severe bronchopulmonary dysplasia: A systematic review and Meta-Analysis. Front. Pediatrics.

[18] Gibbs K., Collaco J.M., McGrath-Morrow S.A. (2016). Impact of tobacco smoke and nicotine exposure on lung development. Chest.

[19] Been J.V., Millett C. (2019). Reducing the global burden of preterm births. Lancet Glob. Health.

[20] Wagijo M.A., Sheikh A., Duijts L., Been J.V. (2017). Reducing tobacco smoking and smoke exposure to prevent preterm birth and its complications. Paediatr. Respir..

[21] Lauren Comarda J.E. Maternal Tobacco Exposure Leads to Cytokine Dysregulation in Placental Membranes Stimulated with Lipopolysaccharide. Proceedings of the Pediatric Academic Society.

[22] Jobe A.H., Bancalari E. (2001). Bronchopulmonary dysplasia. Am. J. Respir. Crit. Care Med..

[23] Fajardy I., Moitrot E., Vambergue A., Vandersippe-Millot M., Deruelle P., Rousseaux J. (2009). Time course analysis of RNA stability in human placenta. BMC Mol. Biol..

[24] Davidson L.M., Berkelhamer S.K. (2017). Bronchopulmonary Dysplasia: Chronic Lung Disease of Infancy and Long-Term Pulmonary Outcomes. J. Clin. Med..

[25] Cheong J.L.Y., Doyle L.W. (2018). An update on pulmonary and neurodevelopmental outcomes of bronchopulmonary dysplasia. Semin. Perinatol..

[26] Tadesse S., Luo G., Park J.S., Kim B.J., Snegovskikh V.V., Zheng T., Hodgson E.J., Arcuri F., Toti P., Parikh C.R. (2011). Intra-amniotic infection upregulates neutrophil gelatinase-associated lipocalin (NGAL) expression at the maternal-fetal interface at term: Implications for infection-related preterm birth. Reprod. Sci..

[27] Lee J., Taneja V., Vassallo R. (2012). Cigarette smoking and inflammation: Cellular and molecular mechanisms. J. Dent. Res..

[28] Gonçalves R.B., Coletta R.D., Silvério K.G., Benevides L., Casati M.Z., da Silva J.S., Nociti F.H. (2011). Impact of smoking on inflammation: Overview of molecular mechanisms. Inflamm. Res..

[29] Yoon B.H., Romero R., Jun J.K., Park K.H., Park J.D., Ghezzi F., Kim B.I. (1997). Amniotic fluid cytokines (interleukin-6, tumor necrosis factor-alpha, interleukin-1 beta, and interleukin-8) and the risk for the development of bronchopulmonary dysplasia. Am. J. Obstet. Gynecol..

[30] Shimoya K., Moriyama A., Matsuzaki N., Ogata I., Koyama M., Azuma C., Saji F., Murata Y. (1999). Human placental cells show enhanced production of interleukin (IL)-8 in response to lipopolysaccharide (LPS), IL-1 and tumour necrosis factor (TNF)-alpha, but not to IL-6. Mol. Hum. Reprod..

[31] Nair R.R., Khanna A., Singh K. (2013). Role of inflammatory proteins S100A8 and S100A9 in pathophysiology of recurrent early pregnancy loss. Placenta.

[32] Isayama T., Shah P.S., Ye X.Y., Dunn M., Da Silva O., Alvaro R., Lee S.K. (2015). Adverse Impact of Maternal Cigarette Smoking on Preterm Infants: A Population-Based Cohort Study. Am. J. Perinatol..

[33] Spinillo A., Ometto A., Stronati M., Piazzi G., Iasci A., Rondini G. (1995). Epidemiologic association between maternal smoking during pregnancy and intracranial hemorrhage in preterm infants. J. Pediatrics.

